# A comparative study of low energy radiation response of AlAs, GaAs and GaAs/AlAs superlattice and the damage effects on their electronic structures

**DOI:** 10.1038/s41598-018-20155-0

**Published:** 2018-01-31

**Authors:** M. Jiang, H. Y. Xiao, S. M. Peng, G. X. Yang, Z. J. Liu, X. T. Zu

**Affiliations:** 1 School of Physics, University of Electronic Science and Technology of China, Chengdu, 610054 China; 2 Institute of Nuclear Physics and Chemistry, Chinese Academy of Engineering Physics, Mianyang, 621900 China; 3 Department of Physics, Lanzhou City University, Lanzhou, 730070 China

## Abstract

In this study, the low energy radiation responses of AlAs, GaAs and GaAs/AlAs superlattice are simulated and the radiation damage effects on their electronic structures are investigated. It is found that the threshold displacement energies for AlAs are generally larger than those for GaAs, i.e., the atoms in AlAs are more difficult to be displaced than those in GaAs under radiation environment. As for GaAs/AlAs superlattice, the Ga and Al atoms are more susceptible to the radiation than those in the bulk AlAs and GaAs, whereas the As atoms need comparable or much larger energies to be displaced than those in the bulk states. The created defects are generally Frenkel pairs, and a few antisite defects are also created in the superlattice structure. The created defects are found to have profound effects on the electronic properties of GaAs/AlAs superlattice, in which charge transfer, redistribution and even accumulation take place, and band gap narrowing and even metallicity are induced in some cases. This study shows that it is necessary to enhance the radiation tolerance of GaAs/AlAs superlattice to improve their performance under irradiation.

## Introduction

In the past decades, the development in micro-fabrication such as molecular beam epitaxy (MBE) and metal organic chemical vapor deposition (MOCVD) opens a new stage in science where artificial materials are designed for specific studies and applications^1^. It is noted that gallium arsenide (GaAs) and aluminum arsenide (AlAs) are perfectly lattice matched, and few difficulties are expected in the growth of (GaAs)_m_/(AlAs)_n_ semiconductor superlattice (SL), which consists of m monolayers of GaAs alternating with n monolayers of AlAs. The artificial SL has been widely used in different applications like the optoelectronic devices with quantum cascade laser, high-frequency oscillators and thermoelectric devices^2–9^, due to the new physical phenomena such as quantum confinement, Brillouin-zone folding and the obtaining of a direct-gap superlattice from their indirect-gap constitutes^10^. In the application field of military and aerospace, the semiconductor materials are exposed to different radiation environments, which may result in defect generation, migration and aggregation, and ultimately may deteriorate their optical and electronic properties and influence their performance which may lead to permanent failure^10–15^. For example, Tanaka *et al*. reported that the photoluminescence intensity and the two-dimensional electron gas mobility of GaAs/AlGaAs heterostructures decreased obviously under electron irradiation^14^. Therefore, it is of great importance to study their phase stability and the radiation damage effects on the electronic properties of the semiconductor materials.

The radiation damage effects of GaAs and AlAs have been extensively studied^16–21^. Wesch *et al*. compared the radiation responses of GaAs and AlAs, and they found the AlAs behaves more robustly under Au^+^ ion irradiation^18^. Sayed *et al*. investigated the low energy displacement events of GaAs and AlAs employing the molecular dynamics (MD) method, who found that the threshold displacement energies (E_d_s) for Al atoms are significantly higher than those for Ga atoms along certain directions^19^. Nordlund *et al*. predicted that interstitials are dominant isolated defects in GaAs under He^+^ ion irradiation^21^. By contrast, the radiation responses of (GaAs)_m_/(AlAs)_n_ SL have received relatively scant attentions^22–24^. Cullis *et al*. irradiated the AlAs/GaAs heterostructures with Si^+^ ions, and they suggested that the AlAs resisted ion damage accumulation far more strongly than the GaAs^22^. Jenčič *et al*. have studied the radiation responses of Al_x_Ga_1−x_As/GaAs (x = 0.2 and 0.85) samples to Kr^+^ and Xe^+^ irradiation and reported that the AlGaAs is more resistant to amorphization than GaAs and the resistance increases with the increasing Al content^24^. In spite of these experimental investigations, no theoretical simulation of dynamic process of radiation damage of GaAs/AlAs SL has been reported in the literature thus far. There still lacks an atomic-level understanding of the micro-structural evolution and the underlying mechanism for defect generation in the semiconductor superlattices.

**Figure 1:**
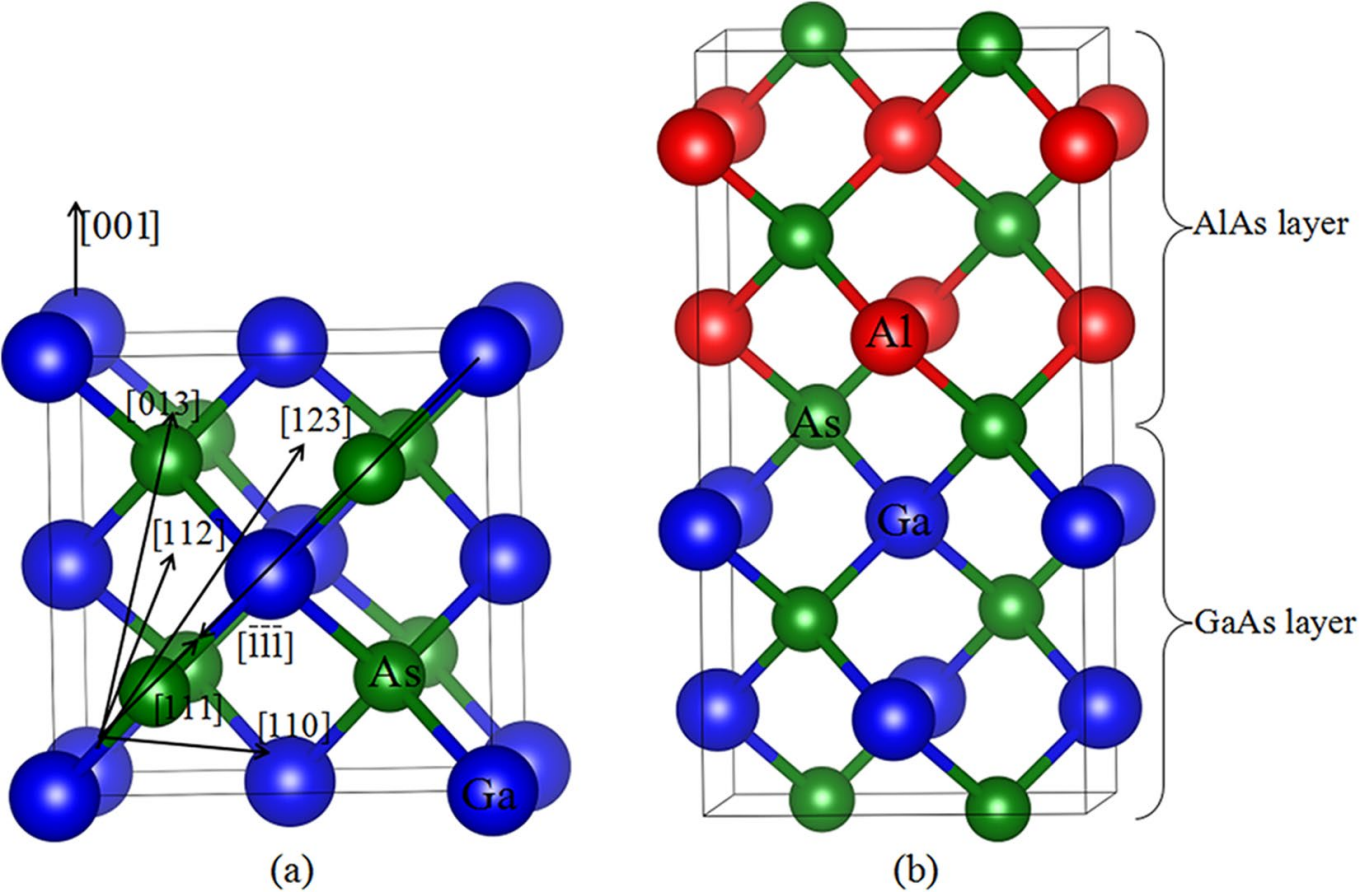
Illustration of schematic view of (**a**) GaAs; (**b**) GaAs/AlAs superlattice. The blue, red and green spheres represent the Ga, Al and As atoms, respectively.

In recent years, the *ab initio* MD (AIMD) method has made it possible to simulate radiation damage of materials with the inclusion of motion of electrons, and has been extensively applied to simulate the displacement events in ceramic and semiconductors materials^25–33^. As compared with the classical MD method, the interatomic potentials are obtained from electronic structure calculations rather than empirical fitting of experimental results. Consequently, a lot of physical parameters like E_d_s can be determined with *ab initio* accuracy. Lucas and Pizzagalli have demonstrated that the average E_d_s for both C and Si sublattices in SiC determined from AIMD are in very close agreement to the experimental consensus and such an agreement has never been obtained with semi-empirical potentials or tight-binding methods^30^. Gao *et al*. carried out AIMD simulation of ion-solid interactions in SiC and revealed that during the dynamic process of displacement events a significant charge transfer occurs between atoms, and the charge transfer to and from recoiling atoms can alter the energy barriers and dynamics for stable defect formation^34^. Wang *et al*. investigated the radiation responses of pyrochlores to electron irradiation using the AIMD method and predicted a number of new mechanisms for defect generation and new defective states that are different from classical MD simulations^33^. These simulations have demonstrated that the AIMD method is a powerful tool in describing the ion-solid interactions in materials. In this study, the AIMD methods are employed to investigate the response behaviors of AlAs, GaAs and GaAs/AlAs SL under low energy irradiation. The geometrical configurations of AlAs, GaAs and GaAs/AlAs SL are illustrated in Fig. 1. The computational details are described in the Methods section. The threshold displacement energies have been determined, and the defect distribution and the pathway for defect generation have been provided. Meanwhile, the radiation damage effects on the electronic structures of these materials have also been investigated. The presented results provide a fundamental insight into the microscopic mechanism of displacement events in AlAs, GaAs and GaAs/AlAs SL, and advance the understanding of the electronic properties of these materials under radiation environment.

## Results and Discussion

### Lattice constants and cohesive energies for bulk AlAs and GaAs

The original point group of AlAs and GaAs crystal is the T_d_ group of zinc blende^35^, as shown in Fig. 1(a). The lattice constants and cohesive energies for AlAs and GaAs are calculated and compared with the available results in Table 1. Our calculated lattice constant of 5.66 Å for AlAs is slightly smaller than the value of 5.71 Å for GaAs, which is in good agreement with the experimental results^36^ reported by Wyckoff and other theoretical results^7,37,38^. In this study, the lattice constant of GaAs/AlAs SL is set to be the intermediate value of 5.685 Å due to the small lattice mismatch between GaAs and AlAs.

**Table 1:** The calculated and experimental structural and energetic properties for bulk GaAs and AlAs.

	AlAs		GaAs	
	a_0_ (Å)	E_coh_ (eV/atom)	a_0_ (Å)	E_coh_ (eV/atom)
Our Cal.	5.66	3.74	5.71	3.21
Other Cal.	5.64^a^ 5.63^b^	4.1^d^ 3.74^e^	5.66^a^, 5.65^c^	3.7^d^ 3.18^e^
Exp.	5.66^f^ 5.62^g^	3.85^h^	5.65^h^ 5.66^g^	3.35^h^

The E_coh_ and a_0_ refer to the cohesive energy and the lattice constant, respectively.

^a^Ref.^7^.

^b^Ref.^37^.

^c^Ref.^38^.

^d^Ref.^41^.

^e^Ref.^40^.

^f^Ref.^48^.

^g^Ref.^36^.

^h^Ref.^39^.

The cohesive energy is the condensed-matter analog of molecular atomization energy and a measure of the inter-atomic bond strength, which is calculated by ${E}_{coh}(AB)=[n\times {E}_{iso}(A)+m\times {E}_{iso}(B)-{E}_{total}(AB)]/(n+m)$. Here, n and m denote the total number of A and B atoms in the unit cell, respectively, *E*_*toatl*_(*AB*) represents the energies of GaAs and AlAs, and *E*_*iso*_(*A*) and *E*_*iso*_(*B*) are the total energies of isolated A and B atoms, respectively. The cohesive energies of AlAs and GaAs are determined to be 3.74 and 3.21 eV/atom, respectively, which are in good agreement with the experimental results reported by Cohen *et al*.^39^ and the theoretical results reported by Ahmed *et al*.^40^. It is shown that our results are smaller than the calculated results of Ihm *et al*.^41^, which is resulted from the different computational details. In the study of Ihm *et al*., the nonlocal (angular-momentum-dependent) pseudopotentials were employed, and the exchange-correlation potential was described by the local-density approximation (LDA) within Winger parameterization^41^, while in our study norm-conserving Troullier-Matrins pseudopotentials are employed, and the LDA method within Ceperly-Alder parameterization is used to describe the exchange-correlation potential. We also find that the cohesive energy of AlAs is larger than that of GaAs, i.e, the <Al-As> bond is stronger than the <Ga-As> bond, which may be partially responsible for their different radiation tolerance.

### Threshold displacement energies in AlAs, GaAs and GaAs/AlAs superlattice

The threshold displacement energy (E_d_), which is defined as the minimum transferred kinetic energy for the primary knock-on atom (PKA) to be permanently displaced from its lattice site, is one of the critical physical parameters for estimating damage production rates and predicting the defect profile under electron, neutron and ion irradiation. In this study, the E_d_s for Al, Ga and As PKAs in bulk AlAs, GaAs and GaAs/AlAs SL are determined and summarized in Tables 2 and 3. For bulk AlAs and GaAs, the MD results reported by Sayed *et al*.^19^ are also included in Table 2 for comparison. Comparing our results with the MD results, we find that the E_d_s obtained by the AIMD method are much smaller in most cases, except for As[110], Al[001], Al[111] and Ga[001]. Previous AIMD simulation of ion-solid interactions in SiC revealed that the displacement event is actually a charge-transfer process and the charge transfer to and from recoiling atoms can alter the energy barriers and dynamics for stable defect formation^34^. The lower values of E_d_ found by AIMD compared to those determined by classical MD may be due to the fact that charge transfer that occurs during the recoil events is taken into account by the AIMD method, while in the classical MD simulation the charge of atoms is fixed.

**Table 2:** Calculated threshold displacement energy (E_d_) for Al, Ga and As recoils in bulk AlAs and GaAs.

Direction	E_d_ (eV)			
	AlAs		GaAs	
	Al PKA	As PKA	Ga PKA	As PKA
[001]	20,14^a^	13.5,16^a^	14.5,14^a^	10,16^a^
[110]	**13**,20^a^	21.5,18^a^	12,16^a^	10,20^a^
[111]	23,22^a^	**13**,18^a^	12,16^a^	**8.5**,16^a^
$[\overline{1}\overline{1}\overline{1}]$	39	32.5	**8**	20
[013]	29	22.5	17.5	12
[112]	17.5	17	22.5	10
[123]	37	**13**	30	10

The minimum values for PKAs are indicated in bold.

^a^Ref.^1^.

**Table 3:** Calculated threshold displacement energy (E_d_) for Al, Ga and As recoils in GaAs/AlAs superlattice.

Direction	E_d_ (eV)		
	As PKA	Al PKA	Ga PKA
[001]	15	33	18
[110]	17.5	15	14
[111]	30	10	9.5
$[\overline{1}\overline{1}\overline{1}]$	12	22.5	17.5
[013]	17.5	20	22.5
[112]	24	17	11
[123]	15	17	12

For AlAs, the minimum E_d_ value is 13 eV for Al PKA along the [110] direction, and the As PKAs are more easily to be displaced along the [111], [123] and [001] directions, as indicated by the small values of 13 and 13.5 eV. Comparing the E_d_ values for Al and As atoms, we find that the As atoms are generally more easily to be displaced than Al atoms, except the case of [110] where the energy of 21.5 eV for As atom is 8.5 eV larger than that for Al atom. These results show that the As displacement may be dominant in the recoil events of AlAs.

For GaAs, the minimum E_d_ for Ga PKA is 8 eV along the $[\overline{1}\overline{1}\overline{1}]$ direction, which is comparable to the minimum value of 8.5 eV for As PKA along the [111] direction. Similar to AlAs, the As atoms are generally more easily to be displaced, since their E_d_ values are generally smaller than those for Ga PKAs, except the case of $[\overline{1}\overline{1}\overline{1}]$. Pons and Bourgoin found that the damaged defects in GaAs were generally caused by displacements of As atoms under electron irradiation^17^, which is consistent with our results. It is noted that for each recoil event the E_d_ values for AlAs are generally larger than those for GaAs, except for the cation recoils along the [112] direction. These results indicate that the lattice atoms in AlAs are generally more difficult to be displaced than those in GaAs under low energy irradiation, which may be caused by larger binding energies of AlAs. Sayed *et al*. investigated low energy displacement events in GaAs and AlAs employing the MD method, who found that the values of E_d_s for Al atoms are significantly higher than those for Ga atoms in certain directions^19^. Wesch *et al*. compared the damage formation of GaAs and AlAs under Au^+^ ion irradiation^18^. Their results showed that the backscattered yield in the region of GaAs increased slightly stronger with the ion fluences than AlAs, which is also in good agreement with our study.

As shown in Fig. 1(b), the GaAs/AlAs SL is terminated by the As layer and the Ga, Al and As atoms on the boundary of the As interface are selected as the PKA. As can be seen from Table 3, the maximum and minimum E_d_ values for the As PKAs are along the [111] and $[\overline{1}\overline{1}\overline{1}]$ directions, respectively, and the respective E_d_ values are 30 and 12 eV. In these two cases, the radiation damage end states show different character. For As[111], the As PKA moves away from its lattice site to eject its neighboring Al atom and occupies an interstitial site (As_int_). The collided Al atom moves along the [111] direction and occupies another nearby As lattice site to form an antisite defect (Al_As_). Then, the third ejected As atom occupies its neighboring Ga lattice site (As_Ga_), and the Ga atom forms an interstitial defect (Ga_int_). In the end, one pair of As FP, one Al vacancy (V_Al_), one Al_As_ antisite defect, one As_Ga_ antisite defect and one Ga_int_ defects are created. The associated defects in the case of As $[\overline{1}\overline{1}\overline{1}]$ are relatively simple, which consist of only one As vacancy (V_As_) and one As_int_. For Al PKAs, the maximum and minimum E_d_ values are determined to be 33 eV along the [001] direction and 10 eV along the [111] direction, respectively, and the pathway for defect generation are very different from each other. In the case of Al[001], besides the Al PKA, a number of neighboring Al and Ga atoms are also involved in the displacement events, which results in the formation of one pair of Al FP, one pair of Ga FP and two Ga_Al_ and Al_Ga_ antisite defects after recoil events. The displacement event in the case of Al[111] is much simpler, and only one pair of Al FP is created. Similarly, the Ga PKA is easy to be displaced along the [111] direction, as indicated by the minimum value of 9.5 eV. It is noted that the E_d_ values of Ga PKAs are generally smaller than those of the Al atoms, except the [013] direction, indicating that the Ga atoms are more easily to be displaced than Al atoms.

**Figure 2:**
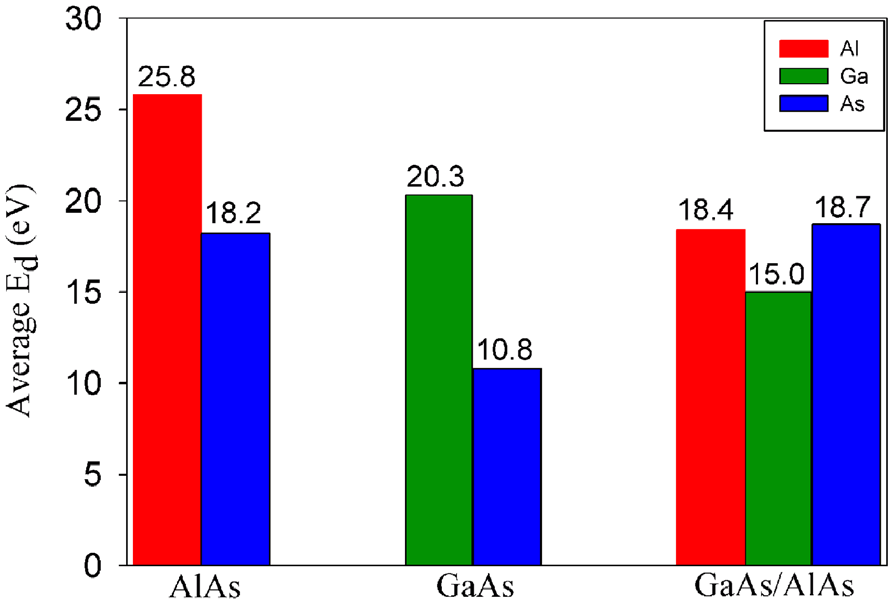
The average threshold displacement energy (E_d_) for Al, Ga and As atoms in bulk AlAs, bulk GaAs and GaAs/AlAs superlattice.

The average threshold displacement energies for PKAs in bulk GaAs and AlAs as well as GaAs/AlAs SL are plotted in Fig. 2. The average E_d_s over all the crystallographic directions considered in this study are calculated by $${E}_{ave}=({E}_{[100]}\times {n}_{1}+{E}_{[110]}\times {n}_{2}+{E}_{[111]}\times {n}_{3}+{E}_{[\overline{1}\overline{1}\overline{1}]}\times {n}_{4}$$ + $${E}_{[013]}\times {n}_{5}+{E}_{[112]}\times {n}_{6}$$ + $${E}_{[123]}\times {n}_{7})/$$$$({n}_{1}+{n}_{2}+{n}_{3}+{n}_{4}$$ + *n*_5_ + *n*_6_ + *n*_7_ where *n*_1_, *n*_2_, *n*_3_, *n*_4_, *n*_5_, *n*_6_ and *n*_7_ are the number of equivalent directions for a specific direction. In bulk GaAs and AlAs, the average E_d_ values for cation recoils are obviously larger than that for As atoms, indicating that As displacement may be the dominant. We also find that the average value of 25.8 eV for Al PKAs is larger than the value of 20.3 eV for Ga PKAs. Although the Ga atom has larger atomic radius and mass than the As atom, the <Ga-As> bond is weaker than the <Al-As> bond, as indicated by the lower cohesive energy of GaAs in Table 1. On the other hand, the screening of the Coulomb force between the Ga PKA and its neighbors is more effective and the interaction between them is relatively smaller, which decreases the energy barrier for defect generation. Consequently, the Ga atoms are relatively more easily to be displaced than the Al atoms. Besides, we find that higher energies are needed for the As atoms in AlAs to be displaced than those for As atoms in GaAs. These results indicate that the AlAs may behave more robustly than the GaAs under radiation environment, agreeing well with theoretical^19^ and experimental^18^ findings. As for GaAs/AlAs SL, the average E_d_ values are determined to be 18.4, 15.0 and 18.7 eV for Al, Ga and As atoms, respectively. It is shown that the cations in the SL structure are more susceptible to the radiation than those in the bulk state, as indicated by the decreased average value of E_d_s. Under electron irradiation the maximum energy transferred to an atom can be expressed as $$T=2{E}_{e}({E}_{e}+2{m}_{e}{c}^{2})/M{c}^{2}$$, where *E*_*e*_ is the incident energy, *m*_*e*_ is the electronic mass, *M* is the atomic mass and *c* is the velocity of light^27^. The E_d_ values of 20.3 and 15 eV for Ga recoil in bulk GaAs and SL correspond to 898 and 815 keV electron irradiation, respectively. As for Al recoil, the E_d_ values of 25.8 and 18.4 eV in bulk AlAs and SL correspond to 715 and 651 keV electron irradiation, respectively. It is noted that the cations in bulk state behave more robustly than those in the SL under electron irradiation. Bryant and Cox have irradiated the samples of CdS and CdTe employing the electron irradiation, and found that the CdS is more resistant to electron irradiation than the CdTe, due to the larger E_d_ value of ~9.6 eV for S atoms than the value of ~7.9 eV for Te atoms^42^. As for the As atoms in the SL structure, the E_d_s are comparable with those for As atoms in the bulk AlAs, while these values are much larger than those for As atoms in bulk GaAs. The similar average E_d_s for As atoms between bulk AlAs and the SL structure may due to the anisotropy of E_d_, which leads to different contributions to the average value from directions with different E_d_ values. Obviously, the GaAs/AlAs SL exhibits different radiation tolerance from the bulk GaAs and AlAs. Comparing the two compositions in the SL structure, we find that the average E_d_s for GaAs are lower than those for AlAs, i.e., GaAs is more susceptible to low energy irradiation. Cullis *et al*. found that the GaAs layer of AlAs/GaAs epitaxial heterostructures was relatively easily amorphized under Si^+^ irradiation, whereas the AlAs layer was quite resistant to damage accumulation and remained its crystalline for the ion doses employed^22^, which agrees well with our results.

### Defect distribution in AlAs, GaAs and GaAs/AlAs superlattice after recoil events

The created defects after each recoil event in bulk AlAs and GaAs are summarized in Tables 4 and 5, respectively. For Al PKAs, the damage end states are generally Al vacancy (V_Al_) and Al interstitial (Al_int_) except the case of Al[123], where the Al occupying the As lattice site (Al_As_) and the As occupying the Al lattice site (As_Al_) defects are created. Although the number of associated defects for Al PKAs is identical, the displacements of the PKA are very different from each other, due to the different defect generation mechanisms. As for the As PKAs in AlAs, the created defects are generally As vacancy (V_As_) and As interstitial (As_int_). It is noted that damage end states in the cases of [110], $[\overline{1}\overline{1}\overline{1}]$ and [123] show different character. For As[110], the As PKA moves 3.68 Å to replace its neighboring Al lattice site (As_Al_), and the collided Al atom occupies the original site of As PKA (Al_As_). In the recoil event of As[123], the As atom moves 1.89 Å away to eject its neighboring Al atom and occupies an interstitial site in the end. The collided Al atom also occupies an interstitial site, which is 1.67 Å away from its lattice site. Consequently, one pair of Al FP and one pair of As FP defects are created after the recoil event. The associated defects for As $[\overline{1}\overline{1}\overline{1}]$ are more complex, where two neighboring Al atoms are involved in the recoil events, and eventually one V_As_ defect, one As_Al_ antisite defect, two Al_int_ defects and one V_Al_ defect are formed. It is noted that the PKA displacements of 5.22 Å for As[001] and 5.20 Å for Al[001] are very close to each other. For the As and Al PKAs along the [001] direction, the first neighboring atoms are As and Al atoms, respectively, and the separating distances between the PKA and its first neighbor in both cases are 5.66 Å, i.e., the lattice constant of bulk AlAs. During the recoil events, the PKA moves along the [001] direction to collide with its first neighboring atom and replace it, which then moves back a little bit due to repulsive interaction. The similar displacements for As and Al PKAs may be due to their similar motion trajectory and similar atomic radii for As and Al, i.e., 1.19 Å for As atom and 1.21 Å for Al atom.

**Table 4:** Defect configuration and the displacement for Al and As PKAs (d_PKA_) in AlAs.

	Al PKA		As PKA	
	Defect type	d_PKA_ (Å)	Defect type	d_PKA_ (Å)
[001]	V_Al_ + Al_int_	5.20	V_As_ + As_int_	5.22
[110]	V_Al_ + Al_int_	4.68	As_Al_ + Al_As_	3.68
[111]	V_Al_ + Al_int_	5.02	V_As_ + As_int_	4.57
$[\overline{1}\overline{1}\overline{1}]$	V_Al_ + Al_int_	1.82	V_As_ + As_Al_ + Al_int_ + V_Al_ + Al_int_	3.33
[013]	V_Al_ + Al_int_	2.32	V_As_ + As_int_	5.14
[112]	V_Al_ + Al_int_	4.18	V_As_ + As_int_	4.32
[123]	Al_As_ + As_Al_	2.42	V_As_ + As_int_ + V_Al_ + Al_int_	1.89

V_Al_: Al vacancy; Al_int_: Al interstitial; V_As_: As vacancy; As_int_: As interstitial; As_Al_: As occupying the Al lattice site; Al_As_: Al occupying the As lattice site.

**Table 5:** Defect configuration and the displacement for Ga and As PKAs (d_PKA_) in GaAs.

	Ga PKA		As PKA	
	Defect type	d_PKA_ (Å)	Defect type	d_PKA_ (Å)
[001]	V_Ga_ + Ga_int_	5.51	V_As_ + As_int_ + V_Ga_ + Ga_int_	2.63
[110]	Ga_As_ + As_Ga_	4.19	V_As_ + As_int_	4.09
[111]	Ga_As_ + As_int_ + V_Ga_	2.08	V_As_ + As_int_	3.94
$[\overline{1}\overline{1}\overline{1}]$	V_Ga_ + Ga_int_	4.87	V_As_ + As_Ga_ + Ga_int_	1.97
[013]	V_Ga_ + Ga_int_	1.85	Ga_As_ + As_int_ + V_Ga_	2.83
[112]	V_Ga_ + Ga_int_	1.84	V_As_ + As_int_	4.18
[123]	2V_Ga_ + 2Ga_int_ + V_As_ + As_int_	4.17	V_As_ + As_int_	4.24

V_Ga_: Ga vacancy; Ga_int_: Ga interstitial; V_As_: As vacancy; As_int_: As interstitial; As_Ga_: As occupying the Ga lattice site; Ga_As_: Ga occupying the As lattice site.

As shown in Table 5, although the created defects for Ga recoil events are generally Ga vacancy (V_Ga_) and Ga interstitial (Ga_int_), the damage end states for Ga[110], Ga[111] and Ga[123] show different character. In the cases of Ga[110] and Ga[111], the Ga atom moves 4.19 and 2.08 Å away to replace its neighboring As lattice site (Ga_As_). In the end, the replaced As atom occupies the Ga PKA lattice site (As_Ga_) for Ga[110], while in the case of Ga[111] the rejected As atom forms the stable interstitial site (As_int_). For Ga[123], one neighboring Ga atom and one As atom are also involved in the recoil events, and the created defects contain two pairs of Ga FP defects and one pair of As FP defect. As for As atoms in GaAs, the associated defects generally consist of V_As_ and As_int_. For As[110], the As PKA leaves its lattice site and moves along the [110] direction. Eventually, it forms a stable interstitial defect, which is 4.09 Å away from its lattice site. The damage end states of the As[001], $[\overline{1}\overline{1}\overline{1}]$ and [013] recoils are somewhat different, where one neighboring Ga atom is involved in the displacement events. As for GaAs, although few antisite defects are created after displacement events, the associated defects are generally vacancies and interstitials. Nordlund *et al*. employed the MD simulations to investigate the atomic-level damage structures in GaAs under ion irradiation and found that a clear majority of the isolated defects produced by low-energy self-recoils and 6 MeV He ions were interstitials^21^.

The created defects after recoil events in GaAs/AlAs SL are summarized in Table 6. For Al atoms, the created defects generally contain V_Al_ and Al_int_, which is similar to the cases of Al PKAs in bulk AlAs. The damage end states for Al[001] and Al $[\overline{1}\overline{1}\overline{1}]$ recoils are somewhat different. For Al $[\overline{1}\overline{1}\overline{1}]$, the Al PKA moves 4.87 Å away and collides its neighboring As atom, and one pair of Al FP and As FP are created in the end. In the case of Al[001], one pair of Al FP, one pair of Ga FP, one Ga occupying the Al lattice site (Ga_Al_) and one Al occupying the Ga lattice site (Al_Ga_) are formed, due to the fact that the other three neighboring atoms are involved in the displacement events. As for Ga atoms, most of the created defects are V_Ga_ and Ga_int_. The recoil events of Ga[001], [110] and $[\overline{1}\overline{1}\overline{1}]$ are found to show different character. In the case of [001], the Ga atom moves away from its lattice site to collide with its neighboring Al atom and occupies the Al lattice site. Then, the ejected Al atom forms an interstitial defect. As for Ga[110], the neighboring As atom is involved in the recoil event, and the radiation damage end states are Ga_As_ and As_Ga_ antisite defects. The associated defects for Ga $[\overline{1}\overline{1}\overline{1}]$ are more complex, as indicated by the created complex defects, i.e., one V_Ga_ defect, one Ga_As_ defect, one As_int_ defect and one pair of Al FP. For the Al and Ga PKAs in GaAs/AlAs SL, the created defects are generally Al FP and Ga FP, respectively, which are similar to the cases of Al atoms in AlAs and Ga atoms in GaAs.

**Table 6:** Defect configuration and the displacement for As, Al and Ga PKAs (d_PKA_) in GaAs/AlAs superlattice.

	Al		Ga		As	
	Defect type	d_PKA_ (Å)	Defect type	d_PKA_ (Å)	Defect type	d_PKA_ (Å)
$[001]$	V_Al_ + Al_int_ + V_Ga_ + Ga_int_ + Ga_Al_ + Al_Ga_	2.57	V_Ga_ + Ga_Al_ + Al_int_	5.20	As_Ga_ + Ga_As_	2.49
[110]	V_Al_ + Al_int_	4.96	Ga_As_ + As_Ga_	4.38	As_Ga_ + Ga_As_	4.17
$[111]$	V_Al_ + Al_int_	4.68	V_Ga_ + Ga_int_	4.30	V_As_ + As_int_ + V_Al_ + Al_As_ + As_Ga_ + Ga_int_	3.95
$[\overline{1}\overline{1}\overline{1}]$	V_Al_ + Al_int_ + V_As_ + As_int_	4.87	V_Ga_ + Ga_As_ + As_int_ + V_Al_ + Al_int_	1.73	V_As_ + As_int_	4.36
$[013]$	V_Al_ + Al_int_	2.31	V_Ga_ + Ga_int_	4.63	As_Ga_ + Ga_As_ + V_Ga_ + Ga_int_	4.13
$[112]$	V_Al_ + Al_int_	6.08	V_Ga_ + Ga_int_	4.91	V_Ga_ + Ga_int_ + Ga_As_ + As_int_ + V_Ga_	3.45
[123]	V_Al_ + Al_int_	4.56	V_Ga_ + Ga_int_	5.07	V_As_ + As_int_	1.61

V_X_: X vacancy (X = Al, Ga and As); X_int_: X interstitial (X = Al, Ga and As); X_Y_: X occupying the Y lattice site (X, Y = Al, Ga and As).

As for the As atoms, the damage end states are somewhat different from those for As PKAs in bulk AlAs and GaAs. The antisite defects are created in each recoil event, except the cases of $[\overline{1}\overline{1}\overline{1}]$ and [123], for which the generated defect is As FP. In the case of [001] and [110], the As_Ga_ and Ga_As_ antisite defects are created, with the PKA displacements of 2.49 and 4.17 Å, respectively, indicative of different defect mechanisms. The associated defects for three other cases are more complex, especially for As[111] recoil, where one pair of As FP, one V_Al_ defect, one Al_As_ defect, one As_Ga_ defect and one As_int_ defect are created. Comparing the defect distributions in AlAs, GaAs and GaAs/AlAs SL, we find that a number of antisite defects are created in GaAs/AlAs SL, whereas very few antisite defects are generated in bulk AlAs and GaAs. However, Frenkel pairs are dominant defects under low energy irradiation in both the bulk and SL structures, which is in good agreement with the results reported by Nordlund *et al*.^21^ and Pronko *et al*.^23^.

### The radiation damage effects on the electronic properties of AlAs, GaAs and GaAs/AlAs superlattice

The (GaAs)_m_/(AlAs)_n_ SLs with (m + n) ranging from 2 to 10 have been widely applied in luminescence and optical absorption, two-phonon absorption and Raman as well as infrared spectra due to their unusual properties^12,43^. Under radiation environment the defect creation, clustering and accumulation may have profound effects on their structural stability and electronic properties, and deteriorate their performance, which may lead to permanent failure. In order to further explore how the radiation damage influences the electronic properties of GaAs/AlAs SL, first-principles calculations based on density functional theory are carried out to investigate the electronic structures of some representative damaged states. The computations are based on a 2 × 2 × 2 supercell consisting of 64 atoms, with a 6 × 6 × 6 k-point sampling in reciprocal space and a cutoff energy of 500 eV.

**Figure 3:**
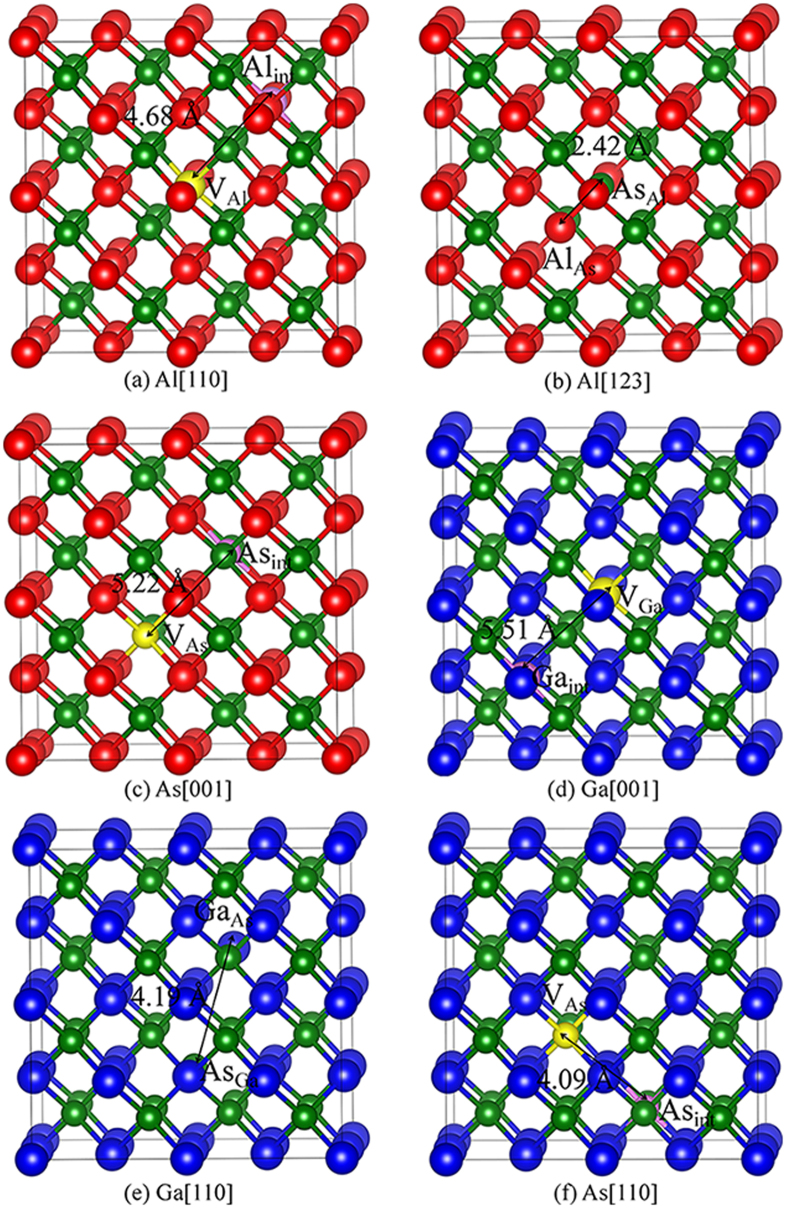
Illustration of schematic view of defects created by PKA recoils in bulk AlAs and GaAs. (**a**) Al[110]; (**b**) Al[123]; (**c**) As[001] in bulk AlAs; (**d**) Ga[001]; (**e**) Ga[110] and (**f**) As [110] in bulk GaAs. The blue, red and green spheres represent the Ga, Al and As atoms, respectively. V_X_: X vacancy (X = Al, Ga or As); X_int_: X interstitial (X = Al, Ga or As); X_Y_: X occupying the Y lattice site (X, Y = Al, Ga or As). The yellow and pink spheres represent the vacancy and interstitial defects, respectively.

**Figure 4:**
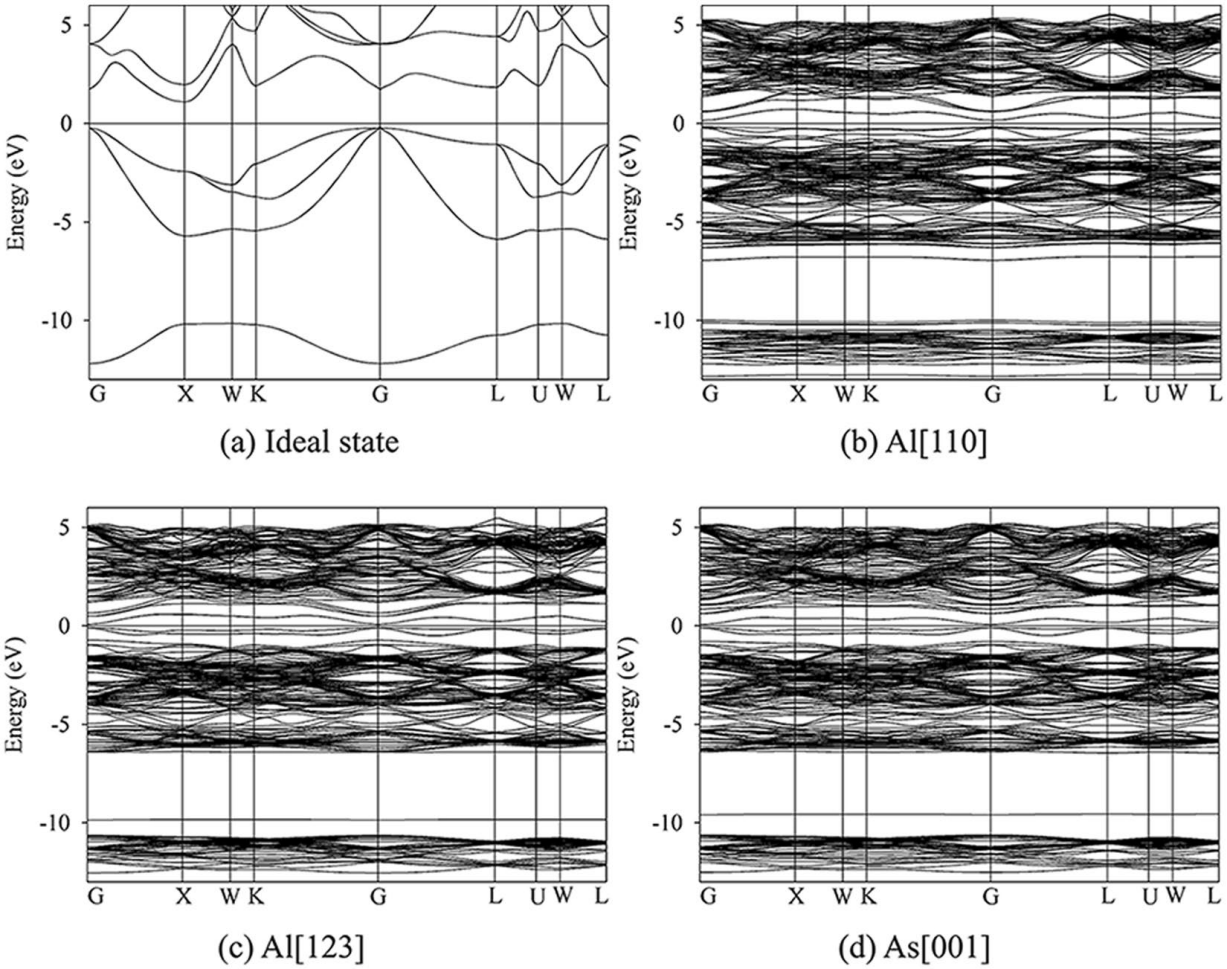
Band structures for ideal and defective AlAs. (**a**) Ideal state; (**b**) Al[110]; (**c**) Al[123] and (**d**) As[001]. Al[110]: Al_int_ and V_Al_ defective state; Al[123]: As_Al_ and Al_As_ antisite defective state; As[001]: As_int_ and V_As_ defective state.

For AlAs and GaAs, the considered defective states are illustrated in Fig. 3(a–c) and (d–f), respectively. The band structures for ideal and defective AlAs are shown in Fig. 4. In Figure 4(a), AlAs has an indirect gap at Χ point, and the energy gap is determined to be 1.31 eV. It is noted that the value of 1.31 eV is consistent with other LDA result of 1.33 eV^5^, whereas it is smaller than the experimental value of 2.16 eV^44^, due to the well-known discontinuity of exchange-correlation energy of the LDA. As one V_Al_ and one Al_int_ defects with separating distance of 4.68 Å are introduced in AlAs, defect levels are observed in the forbidden band region and the energy gap decreases to 0.34 eV, as shown in Fig. 4(b). In the case of Al[123], the Al PKA replaces the As lattice site (Al_As_) and the ejected As atom occupies the PKA lattice site (As_Al_). Figure 4(c) shows that the band gap of antisite-defect state for Al[123] decreases to 0.17 eV. As for As[001], one V_As_ and one As_int_ defects are created, which are separated by 5.22 Å from each other. Similarly, the band gap As-FP-defect state for As[001] is narrowed to 0.15 eV, as illustrated in Fig. 4(d).

**Figure 5:**
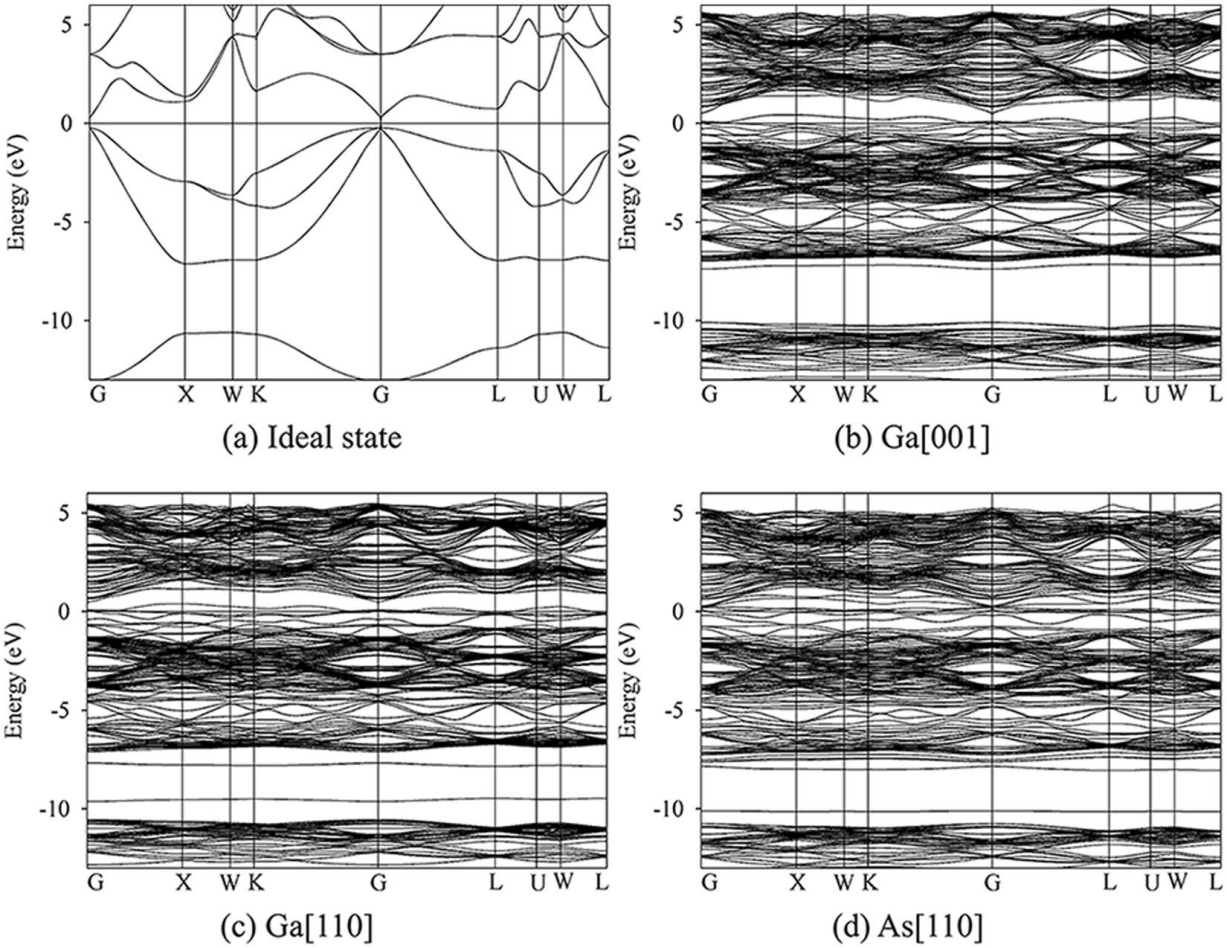
Band structures for ideal and defective GaAs. (**a**) Ideal state; (**b**) Ga[001]; (**c**) Ga[110] and (**d**) As [110]. Ga[001]: Ga_int_ and V_Ga_ defective state; Ga[110]: As_Ga_ and Ga_As_ antisite defective state; As[110]: As_int_ and V_As_ defective state.

Figure 5 describes the band structures for ideal and defective GaAs. Different from AlAs, the energy gap for GaAs is direct with the value of 0.5 eV. This value is comparable with other LDA value of 0.41 eV^15^, while much smaller than the experimental value of 1.52 eV^45^. As show in Fig. 5(b), the defect levels are observed near the valence band maximum (VBM), and cross the Fermi level, indicative of metallic character of defective GaAs with one pair of Ga FP defect. For Ga[110], one pair of Ga_As_ and As_Ga_ antisite defects separated by 4.19 Å are created, and a number of electrons are distributed on the Fermi level, indicative of the metallic character (see Fig. 5(c)). In the case of As[110], the As PKA occupies the interstitial site, which is 4.09 Å away from the lattice site, and one pair of As Frenkel pair defects are created in the end. It is noted that the As[110] defective state also behaves metallic character.

**Figure 6:**
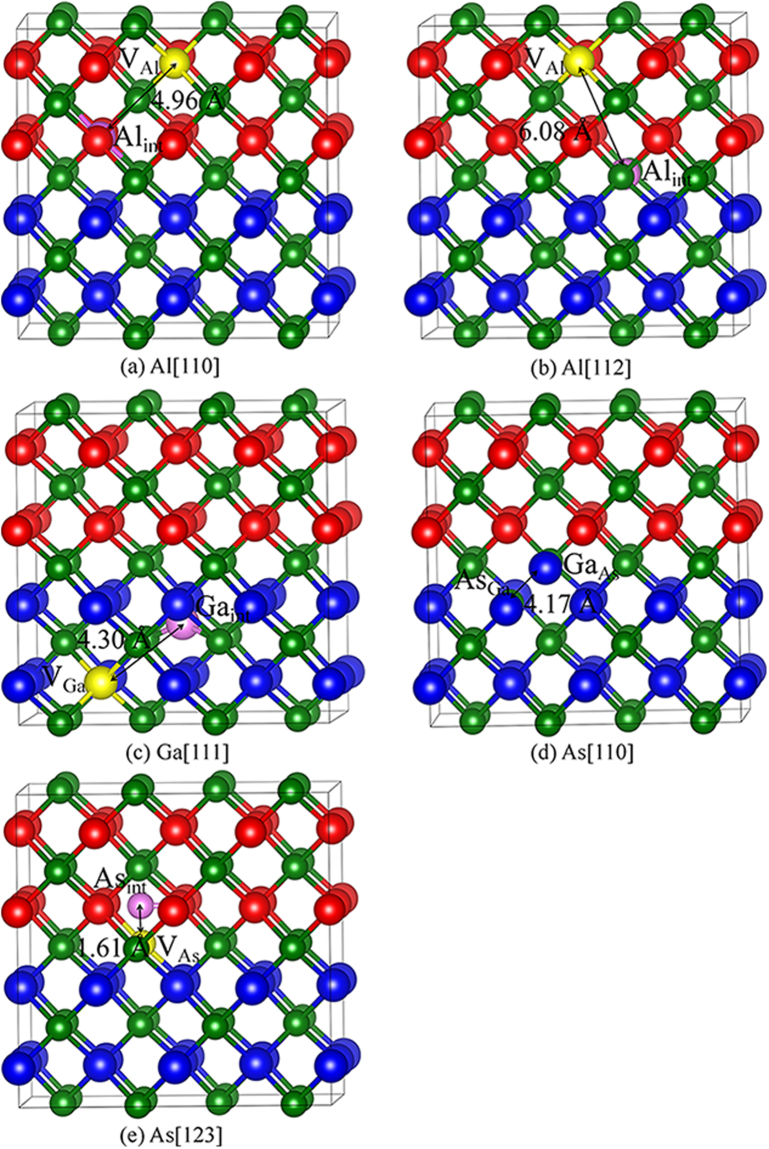
Illustration of schematic view of defects created by PKA recoils in GaAs/AlAs superlattice. (**a**) Al[110]; (**b**) Al[112]; (**c**) Ga[111]; (**d**) As[110] and (**e**) As[123]. The blue, red and green spheres represent the Ga, Al and As atoms, respectively. V_X_: X vacancy (X = Al, Ga or As); X_int_: X interstitial (X = Al, Ga or As); X_Y_: X occupying the Y lattice site (X, Y = Al, Ga or As). The yellow and pink spheres represent the vacancy and interstitial defects, respectively.

**Figure 7:**
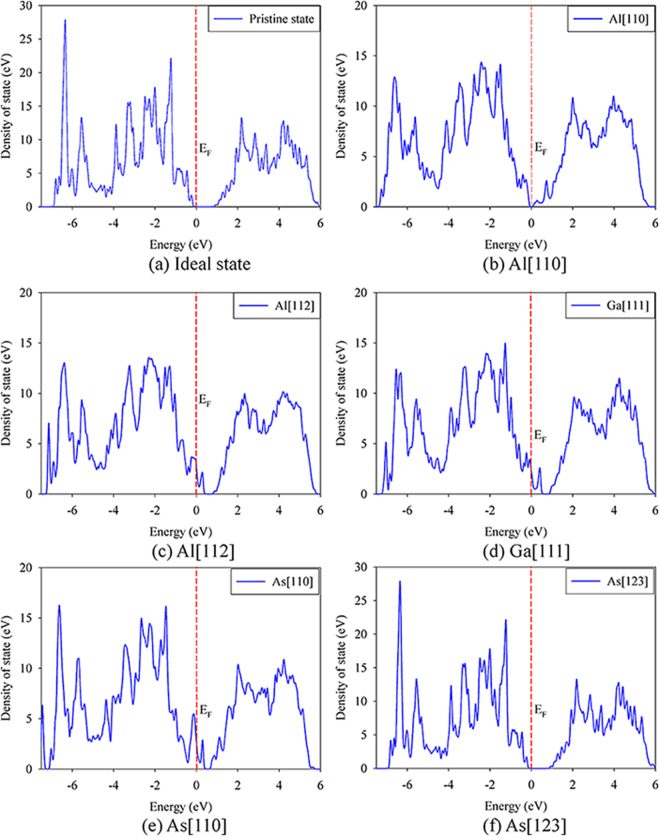
Total density of state distribution for ideal and defective GaAs/AlAs superlattice. The Fermi level is at 0 eV. (**a**) Ideal state; (**b**) Al[110]; (**c**) Al[112]; (**d**) Ga [111]; (**e**) As[110] and (**f**) As[123]. Al[110]: Al_int_ and V_Al_ defective state; Al[112]: Al_int_ and V_Al_ defective state; Ga[111]: Ga_int_ and V_Ga_ defective state; As[110]: As_Ga_ and Ga_As_ antisite defective state; As[123]: As_int_ and V_As_ defective state.

**Figure 8:**
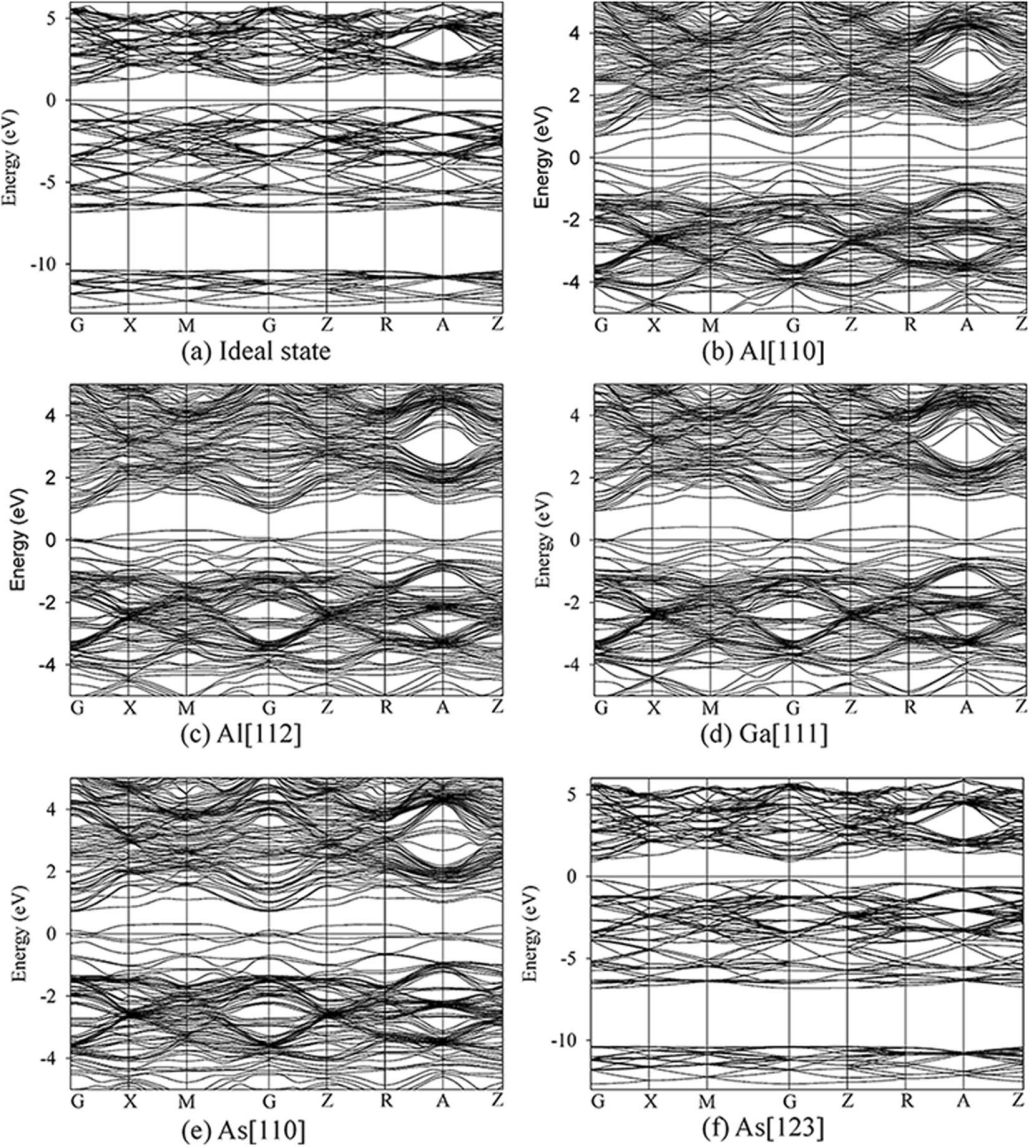
Band structures for ideal and defective GaAs/AlAs superlattice. (**a**) Ideal state; (**b**) Al[110]; (**c**) Al[112]; (**d**) Ga[111]; (**e**) As[110] and (**f**) As[123]. Al[110]: Al_int_ and V_Al_ defective state; Al[112]: Al_int_ and V_Al_ defective state; Ga[111]: Ga_int_ and V_Ga_ defective state; As[110]: As_Ga_ and Ga_As_ antisite defective state; As[123]: As_int_ and V_As_ defective state.

**Figure 9:**
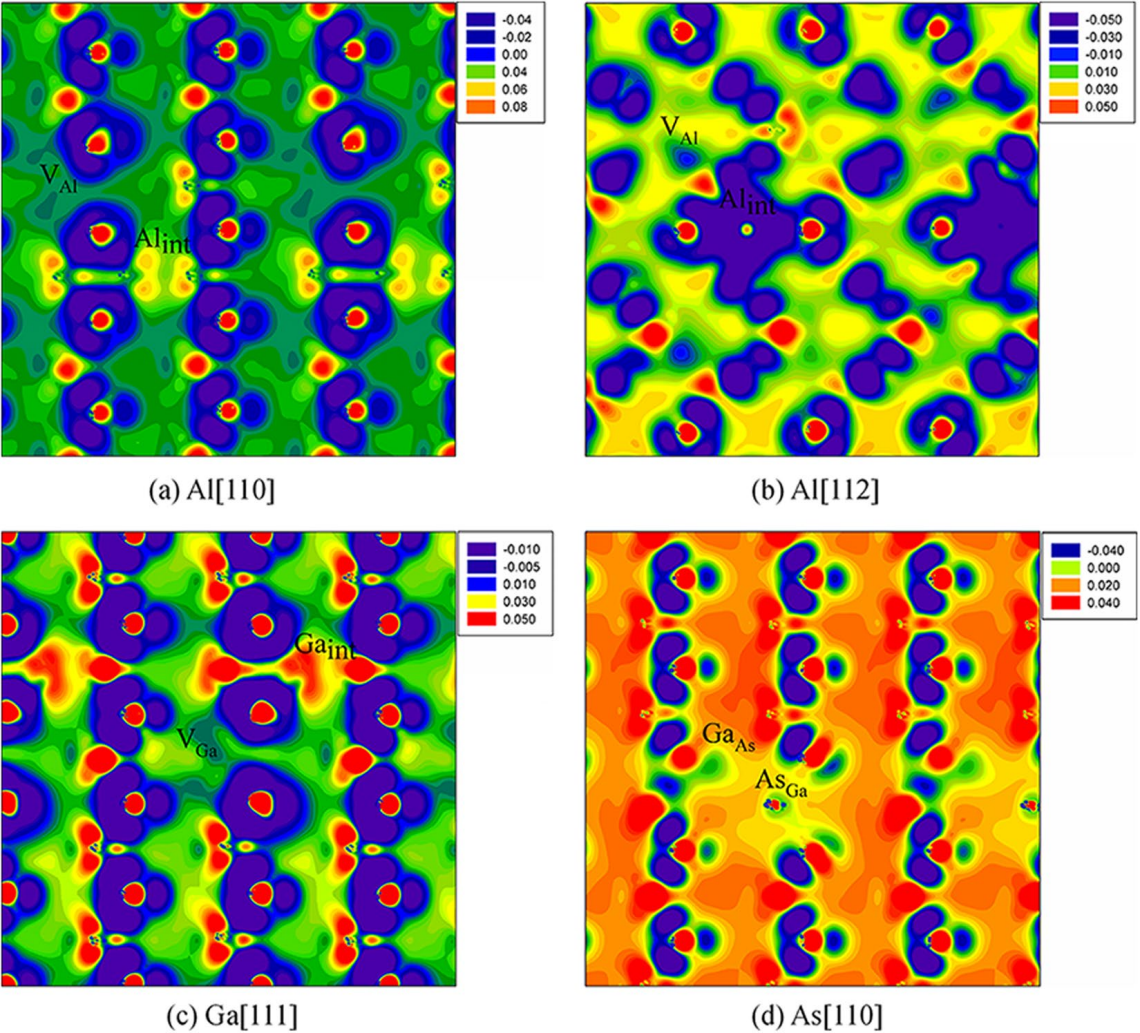
Charge density contour for defective GaAs/AlAs superlattice. (**a**) Al [110]; (**b**) Al[112]; (**c**) Ga[111] and (**d**) As[110]. V_X_: X vacancy (X = Al, Ga or As); X_int_: X interstitial (X = Al, Ga or As); X_Y_: X occupying the Y lattice site (X, Y = Al, Ga or As).

For GaAs/AlAs SL, the damage end states are mainly Frenkel pair and antisite defects. The considered defective states are presented in Fig. 6. The density of state distribution and band structure for ideal and defective SL are illustrated in Figs 7 and 8, respectively. As shown in Fig. 7(a), the energy gap for ideal GaAs/AlAs SL is determined to be 1.14 eV, which is smaller than the experimental value of 2.09 eV^44^, whereas it is in agreement with the theoretical value of 1.16 eV^5^. What is more, the band structure for the ideal SL shows a direct gap at the Γ point, as illustrated in Fig. 8(a). Botti *et al*. have investigated the band structures of (GaAs)_m_/(AlAs)_m_ SL using density functional theory and a semi-empirical method. They suggested that the energy gap for SL is direct at the Γ point when the number of monolayer is no less than 2^5^, which is consistent with our results. In the case of Al[110], the band gap of GaAs/AlAs SL with one pair of Al FP is decreased by about 0.86 eV, as compared with the ideal SL. In Fig. 8(b), the defect levels are observed near the VBM and in the forbidden band region. The charge density difference contour of the SL containing the V_Al_ and Al_int_ defects is illustrated in Fig. 9(a). The Al interstitial forms new bonds with the nearest As atoms, as shown by the charge accumulation in the bonding regions. Figure 9(a) also clearly shows that V_Al_ does not pair with its neighboring atoms, and the Al vacancy is negatively charged. For Al[112], the introduced defects are also Al FP, whereas defective SL behaves metallic character, as confirmed by the density of state distribution (see Fig. 7(c)) and the band structure (see Fig. 8(c)). As shown in Fig. 9(b), significant charge redistribution occurs and electrons are shared by the Al interstitial and its neighboring As atoms. In the case of Ga[111], the introduction of the V_Ga_ and Ga_int_ defects induces the metallicity of GaAs/AlAs SL, due to the fact that the electrons occupy the Fermi level, as shown in the Figs 7(d) and 8(d). The Fig. 9(c) shows the charge density difference of GaAs/AlAs SL with the V_Ga_ and Ga_int_ defects. The Ga interstitial donates electrons to its neighboring As atoms and forms new bonds with them, resulting in electron localization. In the case of As[110], the defect levels appear near the VBM and cross the Fermi level, as shown in Figs 7(e) and 8(e). Figure 9(d) clearly shows that the Ga_As_ and As_Ga_ antisite defects interact with each other and charge delocalization is induced. For the case of As[123], the defect configuration is unstable and the As interstitial recombines with the As vacancy upon structural relaxation. These results suggest that under irradiation the electronic structures of GaAs/AlAs SL are affected significantly, in which charge transfer, redistribution and even accumulation may occur, and band gap narrowing and even metallicity are induced. Consequently, the carrier concentration and mobility, as well as the electrical properties will be influenced. Therefore, it is necessary to enhance the radiation tolerance of GaAs/AlAs SL to improve its electrical performance under radiation environment.

## Conclusions

In summary, low energy recoil events in AlAs, GaAs and GaAs/AlAs superlattice (SL) have been investigated by an *ab initio* molecular dynamics method. The radiation damage effects on the electronic structures of these materials are also studied. In bulk AlAs and GaAs, the threshold displacements energies (E_d_s) for As atoms are generally smaller than those for cations, indicating that the As displacements are dominant in the recoil events of bulk states. Besides, the E_d_ values for AlAs are generally larger than those for GaAs, suggesting that AlAs behaves more robustly under radiation environment. As compared with their bulk states, the cations in GaAs/AlAs SL structure are more susceptible to the radiation, whereas the As atoms are more difficult to be displaced, i.e., the GaAs/AlAs SL exhibits different radiation tolerance from their bulk states. The radiation damage states in bulk AlAs and GaAs are vacancy and interstitial defects. As for GaAs/AlAs SL, the created defects are generally Frenkel pairs (FP) and antisite defects. The band structures of defective GaAs/AlAs SL show that the introduction of FP and antisite defects generally induce metallicity except for the Al FP in the case of Al[110], in which the bandgap of SL decreases by about 0.86 eV, suggesting that the created defects have profound effects on the electronic structures. It is thus necessary to enhance the radiation tolerance of GaAs/AlAs SL to improve its electrical performance under radiation environment.

## Methods

The low-energy displacement events of AlAs, GaAs and (GaAs)_m_/(AlAs)_n_ SL are simulated by the Spanish Initiative for Electronic Simulations with Thousands of Atoms (SIESTA) code. The norm-conserving Troullier-Matrins pseudopotentials^46^ are employed to determine the interaction between ions and electrons, and the exchange-correlation potential is described by the local-density approximation (LDA) in Ceperly-Alder parameterization^47^. The valence wave functions are expanded by a basis set of localized atomic orbitals, and double-ζ basis sets plus polarization orbital (DZP) are employed, with a K-point sampling of 1 × 1 × 1 in the Brillouin zone and a cut-off energy of 60 Ry. The AIMD calculation is computationally expensive and is limited by the system size. However, if the system size is too small, the irradiated atoms will be knocked out of the box. In these cases, a (GaAs)_2_/(AlAs)_2_ superlattice, which consists of two monolayers of GaAs alternating with two monolayers of AlAs (see Fig. 1(b)) and totally 128 atoms, is considered in this work. A specific atom is selected as the primary knock-on atom (PKA), and it is given a kinetic energy to initiate a recoil event. If the PKA returns to its original position at the end of the recoil event, the simulation is restarted at higher recoil energy with an energy increment of 5 eV. Once the PKA is permanently displaced from its lattice site, additional runs are preformed to improve the precision to 0.5 eV. For each atom type, seven principal incidence directions are taken into account in the present study, as shown in Fig. 1(a). The simulations are conducted with an NVE ensemble and the maximum duration of each run is 1.2 ps to avoid the instability of the system.
